# Psychiatric assessment of suicide attempters in Japan: a pilot study at a critical emergency unit in an urban area

**DOI:** 10.1186/1471-244X-7-64

**Published:** 2007-11-07

**Authors:** Tomoki Yamada, Chiaki Kawanishi, Hana Hasegawa, Ryoko Sato, Akiko Konishi, Daiji Kato, Taku Furuno, Ikuko Kishida, Toshinari Odawara, Mitsugi Sugiyama, Yoshio Hirayasu

**Affiliations:** 1 Critical Care and Emergency Center Yokohama City University Medical Center 4-57 Urafune-cho, Minami-ku, Yokohama, 252-0001, Japan; 2 Department of Psychiatry, Yokohama City University School of Medicine 3-9 Fukuura, Kanazawa-ku, Yokohama, 236-0004, Japan

## Abstract

**Background:**

The incidence of suicide has increased markedly in Japan since 1998. As psychological autopsy is not generally accepted in Japan, surveys of suicide attempts, an established risk factor of suicide, are highly regarded. We have carried out this study to gain insight into the psychiatric aspects of those attempting suicide in Japan.

**Methods:**

Three hundred and twenty consecutive cases of attempted suicide who were admitted to an urban emergency department were interviewed, with the focus on psychosocial background and DSM-IV diagnosis. Moreover, they were divided into two groups according to the method of attempted suicide in terms of lethality, and the two groups were compared.

**Results:**

Ninety-five percent of patients received a psychiatric diagnosis: 81% of subjects met the criteria for an axis I disorder. The most frequent diagnosis was mood disorder. The mean age was higher and living alone more common in the high-lethality group. Middle-aged men tended to have a higher prevalence of mood disorders.

**Conclusion:**

This is the first large-scale study of cases of attempted suicide since the dramatic increase in suicides began in Japan. The identification and introduction of treatments for psychiatric disorders at emergency departments has been indicated to be important in suicide prevention.

## Background

In Japan, a dramatic jump in the year-over-year number of suicides occurred in 1998 (32,863 victims compared to 1997 (24,391)); suicide rates have been 25.2–27.0 (per 100,000; from 1998 to 2006) since 1998, whereas, previously, they had remained between 17.0 to 21.0 for over 20 years (from 1978 to 1997). This recent increase has been primarily due to a rise in the number of suicides in middle-aged men, with men in their 50s representing the sharpest peak [1,2]. However, psychiatric and psychosocial aspects of the current trend in suicide increases are not well understood because only one psychological autopsy study was reported before such an increase in Japan [3]. Instead, better insight into individuals' measures against suicide, who unsuccessfully attempted suicide, should improve our understanding, because an unsuccessful attempt is a high-risk factor for subsequent suicide [4-6]; 44% of suicides have a history of unsuccessful attempts [7]. Previous studies indicated that medically serious suicide attempters and suicide victim are two overlapping populations, and share common characteristics [8-10]. Continual care for those that have attempted suicide has the potential for suicide prevention, and the World Health Organization proposed an intervention study of medically treated suicide attempters [11].

This report presents demographic and psychiatric data of patients who suffered serious medical injury as a result of a suicide attempt. It is the first detailed and comprehensive study since the rapid increase in the suicide rate in Japan in 1998. Such data are expected to be crucial if we are to develop effective strategies for high-risk groups regarding suicide.

## Methods

The present study was performed at the Critical Care and Emergency Center, Yokohama City University Medical Center, from April 1, 2003 to September 30, 2005. There are four emergency medical centers in Yokohama, Japan, with a population of 3.5 million people. Our center receives all patients who require critical care from the southern catchment area of the city. During the study period, 2967 patients entered the center, and cases of both suicide attempters and completers numbered 544 (18%). Among these, 320 attempters (126 men and 194 women) who were admitted underwent psychiatric evaluation. The remaining 224 patients did not undergo psychiatric interview because of death on arrival, early death, transfer to another unit with prolonged consciousness disturbances, or an extremely short hospital stay.

The 320 patients were interviewed by at least two trained psychiatrists. Their intent to die was confirmed. Sociodemographics, the method of attempted suicide, current psychiatric treatment, previous suicide attempt/deliberate self-harm, and any family history of suicide were extracted from the interview. Deliberate self-harm was defined as self-destructive behavior without obvious suicidal ideations. Psychiatric diagnosis was made according to the DSM-IV criteria[12] by agreement among more than two psychiatrists (the authors).

Subjects were divided into two groups based on the lethality of the attempt according to the operational criteria of Beautrais [8], and subsequently analyzed. The high-lethality group was defined using the following criteria: 1) mechanical ventilation was required for life support; 2) surgery was performed under general anesthesia; 3) the method of attempted suicide carried a high risk of death, specifically, hanging, gunshot, jumping from a high place, inhalation of gas, solvents, or other agricultural chemicals, thermal injury, or drowning. Data were also stratified according to age. Special attention was given to the high-risk group, males in their 40s and 50s, defined as "middle-age men".

Data are presented as the mean ± SD. Statistical analyses were conducted using SPSS for Windows Version 11.5 (SPSS, Chicago, IL, USA). The chi-square test, Fisher's exact test, and the Mann-Whitney rank sum test were used in comparisons as indicated. A probability level of P < 0.05 was considered significant.

This study was approved by the ethical committee of Yokohama City University School of Medicine.

## Results

### Sociodemographic background (Table 1)

**Table 1:** Sociodemographic and clinical characteristics of cases of suicide attempters

		Male(N = 126)	Female(N = 194)	Analysis		
		N (%)	N (%)	χ	df	P-value
Age						
	< 20	4 (3)	14 (7)			n.s.
	20–29	31 (25)	75 (39)			n.s.
	30–39	29 (23)	46 (24)			n.s.
	40–49	20 (16)	24 (12)			n.s.
	50–59	23 (18)	15 (8)	8.1	1	< 0.01
	60–64	6 (5)	5 (3)			n.s.
	65 ≧	13 (10)	15 (8)			n.s.
	Median	39.0	30.5			
	Mean^§^	42.5 ± 16.0	35.6 ± 15.3	z = -4.1		< 0.001
Stay in hospital (days)						
	Mean^§^	20.2 ± 44.5	16.0 ± 28.5			n.s.
Marital status						n.s.
	Single	56 (44)	75 (39)			
	Married	41 (33)	67 (35)			
	Divorced	16 (13)	22 (11)			
	Widowed	0 (0)	3 (2)			
	Common-law	5 (4)	16 (8)			
Current psychiatric treatment				9.4	2	< 0.01
	Outpatient	63 (50)	141 (73)			
	Hospitalization	2 (2)	2 (1)			
	No treatment	56 (44)	33 (17)			
Living status				13.9	1	< 0.001
	Alone	40 (32)	162 (84)			
	Together	83 (66)	28 (14)			
Education						n.s.
	Compulsory education *	27 (21)	51 (26)			
	High school education and over	79 (63)	128 (66)			
Previous deliberate self harm				11	2	< 0.01
	0	87 (69)	97 (50)			
	1	6 (5)	19 (10)			
	> 2	24 (19)	66 (34)			
Previous suicide attempt				11	2	< 0.01
	0	83 (66)	85 (44)			
	1	19 (15)	48 (25)			
	> 2	15 (12)	48 (25)			
Family history of suicide/attempt		15 (12)	29 (15)			n.s.
Methods						
	Drug over dose	45 (36)	111 (57)	14.1	1	< 0.001
	Laceration	30 (24)	22 (11)	8.7	1	< 0.01
	Jumping from high place	13 (10)	29 (15)	1.4	1	< 0.05
	Poisoning	8 (6)	16 (8)			n.s.
	Inhaling carbon monoxide	11 (9)	2 (1)			
	Burn	6 (5)	3 (2)			
	Traffic death	3 (2)	5 (3)			
	Hanging	4 (3)	3 (2)			
	Drowning	4 (3)	1 (1)			
	Other	2 (2)	2 (1)			
	Firearm	0 (0)	0 (0)			

* Compulsory education lasts for 9 years; statutory schooling ages are between 6 and 14 years in Japan. §Mann-Whitney U test

The mean age of the 320 subjects was 38.3 ± 15.9 years, ranging from 15 to 88 years. Men (42.5 ± 16.0 years) were older than women (35.6 ± 15.3 years) (P < 0.001). Thirty-nine percent of women were in their 20s. Men in their 20s were also represented (25%), followed by the 30s (23%), and 50s (18%). The sum of patients in their 40s and 50s accounted for 34% of men overall.

No significant difference except in living status was observed between genders; the incidence of living alone was significantly higher in women (P < 0.001).

### Method of suicide attempt, previous suicide attempts, and previous, deliberate self harm

The most common method of attempted suicide was drug-overdose in both men and women (36% and 57%, respectively, Table 1). Among the self-poisoners, 75.8% of them used drugs which were prescribed by the out patient clinic. Frequencies of drug-overdosing and jumping from a high place were higher in women than men (P < 0.001 and P < 0.05, respectively), while laceration was more frequent in men (P < 0.01).

Histories of a previous suicide attempt and deliberate self-harm were common, especially in women (previous, deliberate self-harm: 24% in men vs. 44% in women, P < 0.01; previous suicide attempts: 27% in men vs. 50% in women, P < 0.01).

### Psychiatric treatment and diagnosis

Three hundred and three subjects (95%) met the criteria for either an axis I or axis II psychiatric diagnosis or both. Two hundred and sixty subjects (81%) met the criteria for an axis I disorder (Table 2). Mood disorders (24%) were the most common, followed by adjustment disorders (18%), schizophrenic disorders (17%), and substance-abuse related disorders (11%). Among women, adjustment disorders were more common and substance-abuse related disorders were less common than in men (P < 0.05 and P < 0.05, respectively).

**Table 2:** Distribution of suicide by mental disorder and personality disorder

	Male Patients (N = 126)	Female Patients (N = 194)	Analysis			
	N (%)	N (%)	χ^2^	df	P-value	
Axis-I						
Mood disorders	37 (29)	41 (21)	5.78	2	0.056	n.s.
Depressive disorders	33 (26)	38 (20)				
Bipolar disorders	4 (3)	3 (2)				
Adjustment disorders	14 (11)	43 (22)	8.8	2	< 0.05	
Schizophrenia and other psychotic disorders	23 (18)	31 (16)				n.s.
Substance-related disorders	25 (20)	11 (6)	12.2	2	< 0.01	
Anxiety disorders	4 (3)	8 (4)				
Dissociative disorders	0 (0)	6 (3)				
Somatoform disorders	1 (1)	3 (2)				
Dementia	1 (1)	2 (1)				
Eating disorders	0 (0)	3 (2)				
Other axis I diagnosis	4 (3)	3 (2)				
No axis I diagnosis	9 (7)	37 (19)				
Insufficient information for axis I assessment	8 (6)	6 (3)				
Axis II						
Personality disorders	30 (24)	80 (41)	13	2	< 0.01	
Mental retardation	4 (3)	2 (1)				
No axis II diagnosis	84 (67)	106 (55)				
Insufficient information for axis II assessment	8 (6)	6 (3)				

Some subjects had more than one diagnosis. But one primary dignosis only were listed on Axis I and II

One hundred and seven subjects (35%) met axis II criteria for a personality disorder. Personality disorders were more common in women than men (P < 0.01), but 66% of patients with a personality disorder had a concomitant axis I disorder.

Sixty-five percent of all patients were undergoing active psychiatric treatment at the time of the attempt, though women were more likely to be receiving treatment than men (P < 0.01, Table 1).

### High-lethality and low-lethality (Table 3)

**Table 3:** Sociodemographic and clinical characteristics of cases of suicide attempters in the high and low lethality group

		High lethality group (N = 225)	Low lethality group (N = 95)	Analysis		
		N (%)	N (%)	χ^2^	df	P
Gender						n.s.
	Male	96 (43)	30 (32)			
	Female	129 (57)	65 (68)			
	Male middle age	34 (15)	9 (9)	5.9	1	< 0.05
Age						
	Mean^§^	39.8 ± 15.7	34.8 ± 15.9	z = -3.1		< 0.01
Stay in hospital (days)						
	Mean^§^	23.0 ± 41.3	5.0 ± 4.1	z = -8.7		< 0.001
Marital status						n.s.
	Single	87 (39)	44 (46)			
	Married	81 (36)	27 (28)			
	Divorced	31 (14)	7 (7)			
	Widowed	2 (1)	1 (1)			
	Common-law	14 (6)	7 (7)			
Employed status						n.s.
	Employed	95 (42)	38 (40)			
	Unemployed	126 (56)	52 (55)			
Current psychiatric treatment						n.s.
	Outpatient	135 (60)	69 (73)			
	Hospitalization	4 (2)	0 (0)			
	No treatment	71 (32)	18 (19)			
Living status				6.6	2	< 0.05
	Alone	51 (23)	17 (18)			
	Together	172 (76)	73 (77)			
Recent stressful life event						n.s.
	0	73 (32)	22 (23)			
	1	80 (36)	42 (44)			
	> 2	47 (21)	20 (21)			
Education						
	Compulsory education	53 (24)	25 (26)			n.s.
	High school education and over	150 (67)	57 (60)			
Previous deliberate self harm						n.s.
	0	145 (64)	39 (41)			
	1	18 (8)	7 (7)			
	> 2	50 (22)	40 (42)			
Previous suicide attempt						n.s.
	0	121 (54)	47 (49)			
	1	51 (23)	16 (17)			
	> 2	38 (17)	25 (26)			
Family history of suicide/attempt		34 (15)	10 (11)			n.s.
Axis I						n.s.
Mood disorders		58 (26)	20 (21)			
Adjustment disorders		33 (15)	24 (25)			
Schizophrenia and other psychotic disorders		43 (19)	11 (12)			
Substance-related disorders		29 (13)	7 (7)			
Axis II						n.s.
Personality disorders		71 (32)	39 (41)			

§Mann-Whitney U test

Two hundred and twenty-five patients (70%) were assigned to the high-lethality group. Seventy-six percent of men and 66% of women. Patients in the high-lethality group were older [39.8 ± 15.7 years vs. 34.8 ± 15.9 years, respectively, P < 0.01], required a longer hospital stay [23.0 ± 15.7 days vs. 5.0 ± 4.1 days, respectively, P < 0.001], and were more likely to be living alone at the time of the attempt (P < 0.05).

### Characteristics of middle-aged men (Table 4)

**Table 4:** The comparison of "middle age"class and other classes except for "middle age"

		Middle age (n = 43)	except middle age(n = 277)	Analysis		
		N (%)	N (%)	χ^2^	df	P
Methods						
	Drug over dose	11 (26)	145 (52)	10.7	1	< 0.01
	Laceration	13 (30)	39 (14)	7.1	1	0.01
	High lethality	34 (79)	191 (69)	1.8	1	0.21
Education				5.9	2	0.052
	Compulsory education	7 (16)	71 (26)			
	High school education and over	27 (63)	180 (65)			
Axis I						
Mood disorders		16 (37)	62 (22)	4.4	1	0.054
Adjustment disorders		3 (7)	54 (19)	4.0	1	0.053
Previous deliberate self harm		6 (14)	115 (42)	12.1	2	< 0.01
Current psychiatric treatment		23 (53)	185 (67)	8.8	3	< 0.05

Middle-aged men were compared to the other patient groups. Firstly, middle-aged men were over-represented in the high- relative to the low-lethality group, and laceration was more common than in the other groups (P < 0.01), while drug over-dose was less common (P < 0.01). A history of deliberate self-harm was less frequent (P < 0.01). The incidence of mood disorder (37%) tended to be more common (P = 0.054), but active psychiatric treatment was less common in this group (P < 0.05). There were no significant differences between "middle age men" and other men in all items (data not shown).

## Discussion

Suicide attempt is a known potent risk factor. About 0.5% to 2% of individuals complete suicide, and 5% commit suicide within 9 years. [5] Previous studies have found that victims of both successful and failed suicide attempts who suffer medically serious complications share some common characteristics [8-10]. Consequently, evaluation of those attempting suicide can offer: 1. insight into the pathology of successful suicide by providing psychological data, 2. biological studies can be performed, and 3. through prospective studies, the efficacy of interventions can be assessed [13]. In Japan, psychological autopsy studies are not generally accepted. Surveys of failed suicide attempts and interventions for those attempting suicide have been more highly regarded to date.

Few reports have examined psychiatric diagnoses in cases of suicide attempts following the recent rise in the suicide rate in Japan [14-16]. Murase, et al. (2003) studied 100 attempted suicide cases, attempters and found that depressive disorders were the most frequent pathology. Ichimura, et al. (2005) investigated changes in the prevalence of psychiatric diagnoses (ICD-10 classification) between 1992–1993 and 2000 in over 200 attempted suicide cases, and discovered that F20–29 diagnoses had decreased and disorders other than F30–39, F40–48, and F60–69 had also decreased. The present study is the first large-scale study involving a comprehensive evaluation of attempted suicide cases in Japan since the dramatic increase in the suicide rate began.

The most common method of attempted suicide in our subjects was self-poisoning, and the majority of patients who overdosed used psychotropic drugs that had been prescribed. It is known that methods vary between countries and regions. Jumping from a high place seemed more common in our subjects than in previous studies [17-19]. Our center is located in an urban area containing many high-rise office buildings. Therefore, it is possible that there was a selection bias. Conversely, ingestion of pesticides or agricultural chemicals was very rare in our urban population. Attempts using firearms were also rare. Possession of firearms is strictly controlled in Japan, whereas firearm use is the most common method of suicide/attempted suicide in the United States [20].

The overall female/male ratio of cases of attempted suicide was 1.58. This ratio was higher in the low- (2.17) than the high-lethality group (1.34). This is consistent with other reports that women are more likely to attempt suicide than men, but men are more likely to succeed [21,22]. Thus, it seems that patients in the high-lethality group resembled suicide victims, at least from the viewpoint of gender.

Our study was consistent with other reports noting a high prevalence of patients who met the DSM-IV criteria for a psychiatric diagnosis, especially axis I. It has been well established that the vast majority of suicide victims exhibit psychiatric morbidity [23-32]. Bertolote, et al. (2004) compiled psychological autopsy studies, and found that 98% of suicide victims had a diagnosis of at least one mental disorder, with mood disorders accounting for 30.2%, followed by substance -abuse related disorders, and schizophrenia. In our study, 81% of attempted suicide cases had an axis I diagnosis. Mood disorders were the most common, as reported in previous studies which targeted those attempting suicide [33,34]. Thirty-seven percent of all subjects had an axis II diagnosis, and 28% of patients who met the criteria for an axis I diagnosis had concomitant personality disorders. The frequency of personality disorder has been reported to be higher in parasuicide than suicide [35-37]. Engström, et al. (1997) [38] reported that 63% of those attempting suicide met the DSM-III-R criteria for personality disorder. Their subjects were all hospitalized, but whether they suffered from medically serious conditions is unknown.

Middle-aged men are an important subgroup. This population is less likely to have a history of self-harm, but is more likely to use lethal means such as laceration. They are more likely to be depressed, but the prevalence of mood disorders is more common, while they are less likely to seek medical attention, even though the Japanese medical system provides prompt, direct access to a psychiatrist.

### Methodological considerations

The first limitation of our study is that structured interviews were not used to diagnose psychiatric disorders. Generally, hospitalization in our emergency department is too short to perform structured interviews for patients. Instead, psychiatric diagnosis was made by agreement between more than two trained psychiatrists. The second is that we could not fully investigate suicide attempters who did not participate in our study. The third is that our study cohort was limited to an urban-based population. Different outcomes may be expected in a rural-based population. Thus, a multicentre study focusing on multiple sites is required to demonstrate current trends in attempted suicide in Japan.

## Conclusion

This study is the first comprehensive evaluation of consecutive patients admitted to hospitals because of serious medical injury sustained due to a suicide attempt. Our result provides data that can be used to develop and refine strategies for preventing suicide. The present study suggested that introduction and continuation of psychiatric treatment is important for those who had not been treated. Accessibility and availability of psychiatric treatment is a key especially for men. On the other hand, optimal psychiatric treatment should be taken into consideration for those who attempted suicide during outpatient clinic treatment.

## Abbreviations

DSM: The Diagnostic and Statistical Manual of Mental Disorders

ICD: International Statistical Classification of Diseases and Related Health Problems

## Competing interests

The author(s) declare that they have no competing interests.

## Authors' contributions

All authors contributed to study design. TY, RS, HH, AK contributed to data collection. TY, CK, HH, RS wrote the analysis plan, TY analysed data. TY was principal author of the paper, had full access to all data, and is guarantor. All authors contributed to manuscript drafting and revision and approved the final manuscript.

## Pre-publication history

The pre-publication history for this paper can be accessed here:


